# The development of an effective synthetic route of rilpivirine

**DOI:** 10.1186/s13065-021-00749-y

**Published:** 2021-04-01

**Authors:** Tao Zhang, Jiapei Yang, Zhongxia Zhou, Zhipeng Fu, Srinivasulu Cherukupalli, Dongwei Kang, Peng Zhan, Xinyong Liu

**Affiliations:** 1 Department of Medicinal Chemistry, Key Laboratory of Chemical Biology (Ministry of Education), School of Pharmaceutical Sciences, Cheeloo College of Medicine, Shandong University, 44 West Culture Road, Jinan, 250012 Shandong People’s Republic of China; 2 China-Belgium Collaborative Research Center for Innovative Antiviral Drugs of Shandong Province, 44 West Culture Road, Jinan, 250012 Shandong People’s Republic of China

**Keywords:** Rilpivirine, Synthetic optimization, Microwave-promoted method, HIV, Antiviral

## Abstract

**Background:**

Rilpivirine (RPV) was approved by the U.S. FDA (Food and Drug Administration) in 2011 to treat individuals infected with human immunodeficiency virus 1 (HIV-1). Significantly, rilpivirine is three fold more potent than etravirine. Once-daily, it is used with a low oral dose (25 mg/tablet), decreasing the drug administration and bringing a better choice to the patients. However, there are many shortcomings in the existing synthesis route of RPV, such as the high cost, prolonged reaction time and low yield (18.5%).

**Results:**

This article describes our efforts to develop an efficient and practical microwave-promoted method to synthesize rilpivirine using less toxic organic reagents and low boiling solvents. The last step's reaction time decreased from 69 h to 90 min through this optimized synthetic procedure, and the overall yield improved from 18.5 to 21%. In addition, the yield of intermediate **3** increased from 52 to 62% compared to the original patent.

**Conclusion:**

Overall, through a series of process optimization, we have developed a practical synthesis method of rilpivirine, which is easy to scale with higher yield and shorter reaction time.

## Background

**Fig. 1.**
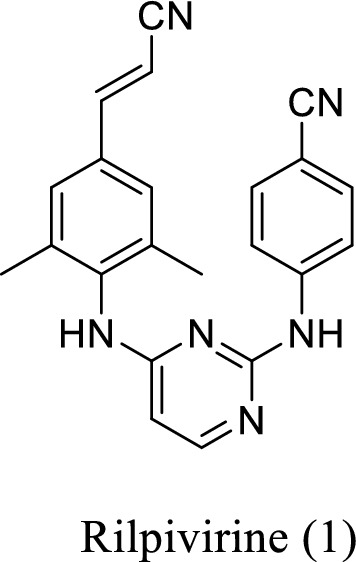
The chemical structure of rilpivirine approved by the U.S. FDA in 2011

HIV-1 non-nucleoside reverse transcriptase (RT) inhibitors (NNRTIs) are an integral part of highly active antiretroviral therapy (HAART) due to its high tolerability and low toxicity. However, the rapid emergence of drug-resistant HIV-1 strains limited their clinical use [1–7]. Rilpivirine ((*E*)-4-((4-((4-(2-cyanovinyl)-2,6-dimethylphenyl)amino)pyrimidin-2-yl)amino)benzonitrile), is a successful second-generation NNRTI of diarylpyrimidine family (Fig. 1), approved by the U.S. FDA in 2011 for the treatment of individuals infected with HIV-1. Rilpivirine has a high genetic barrier to form resistance mutations and generally requires multiple mutations to confer significant resistance [8–10]. Several research-scale synthetic methods have been reported for the preparation of rilpivirine. However, there are some shortcomings among the existing synthetic routes, such as low yield (18.5%), use of high boiling point solvent [(dimethoxyethane (DME), *N*-methylpyrrolidin-2-one (NMP), etc.) and toxic materials (acrylonitrile, etc.,)] which lead to a high price of rilpivirine. Therefore, a new synthetic route is needed urgently for a more economical, less toxic and more productive rilpivirine synthesis.

## Organic synthesis of rilpivirine

The current procedure for the synthesis of rilpivirine is mainly divided into three steps: (a) synthesis of (*E*)-3-(4-amino-3,5-dimethylphenyl)acrylonitrile hydrochloride (intermediate **2**); (b) synthesis of 4-((4-chloropyrimidin-2-yl)amino)benzonitrile (intermediate **3**); (c) synthesis of rilpivirine (**1**).

### Synthesis of (2*E*)-3-(4-amino-3,5-dimethylphenyl)prop-2-enenitrile hydrochloride (intermediate **2**)

**Scheme 1.**
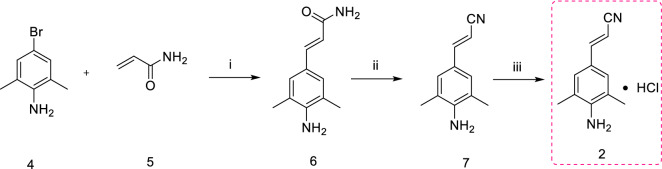
Synthesis of intermediate **2** from 5-bromo-2,4,6-trichloropyrimidine (**4**) and acrylamide (**5**) as starting materials [11]. Reagents and conditions: (i) Pd(OAc)_2_, P(C_6_H_5_CH_3_)_3_, Et_3_N, CH_3_CN, N_2_, 79 °C, overnight, 79.5%; (ii) POCl_3_, 0 °C, 30 min; 20 °C, overnight, 84%; (iii) EtOH, ((CH_3_)_2_CH_2_)_2_O, N_2_, 60 °C, 30 min; HCl, 2-propanol, 60 °C, 30 min, 77%

Scheme 1 illustrates the synthesis of intermediate **2**. Initially, the Heck reaction was performed by allowing the reaction between 4-bromo-2,6-dimethylaniline (**4**) and acrylamide (**5**) under inert conditions to achieve (2*E*)-3-(4-amino-3,5-dimethylphenyl)prop-2-enamide (**6**). Dehydration of compound **6** by treating with POCl_3_ yielded (2*E*)-3-(4-amino-3,5-dimethylphenyl)prop-2-enenitrile (**7**). Finally, the desired intermediate **2** ((2*E*)-3-(4-amino-3,5-dimethylphenyl)prop-2-enenitrile hydrochloride) with 77% yield was achieved by treating compound **7** with HCl in diisopropyl ether under inert condition. The application of palladium acetate (67$/g, 1Pluschem) and tri-*O*-methylphenylphosphine (8$/g, A2B Chem) ligand has greatly improved the yield of *trans*-isomer. However, these two materials are relatively expensive to use in the industry and environmentally polluting [11, 12].

**Scheme 2.**
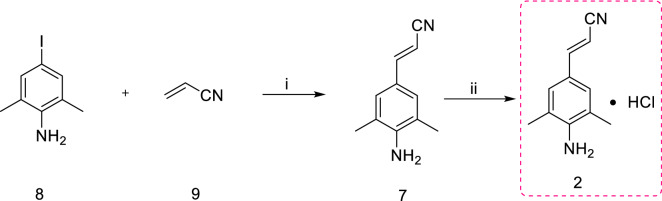
Synthesis of intermediate **2** from 4-iodo-2,6-dimethylaniline (**8**) and acrylonitrile (**9**) as starting materials [13]. Reagents and conditions: (i) CH_3_COONa, Pd/C, DMAC, N_2_, 140 °C, 21 h, 81%; (ii) EtOH, HCl, 2-propanol, 60 °C, 1 h, 64.5%

Scheme 2 also represents the successful synthesis of intermediate **2**, and it differs from Scheme 1 by only starting materials and reagents used in the first step. Starting materials 4-iodo-2,6-dimethylaniline (**8**) and acrylonitrile (**9**) were treated by Heck reaction to obtain compound **7**. Intermediate **2** was obtained by treating compound **7** with HCl in diisopropyl ether under inert condition with 64.5% yield. Although scheme 2 is shorter than Scheme 1 and palladium/carbon is recyclable and cheaper than palladium acetate, the acrylonitrile belongs to B-class organic toxicants. It is too toxic, dangerous and controlled by the public security department, restricting the commercial production [13, 14].

### Synthesis of 4-[(4-chloropyrimidin-2-yl)amino]benzonitrile (intermediate **3**)

**Scheme 3.**
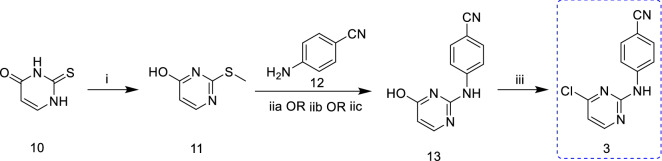
Synthesis of intermediate **3** from 2-thioxo-2,3-dihydropyrimidin-4(1H)-one (**10**) as starting material [15–17]. Reagents and conditions: (i) CH_3_I, NaOH, r.t., overnight, 88%; (iia) DME, reflux, 18 h, 68%; (iib) 180–190 °C, 10 h, 70–74%; (iic) 180 °C, 8 h, 73.6% (iii) POCl_3_, reflux, 20 min, 77%

Scheme 3 represents the chemical pathway for the preparation of intermediate **3** (4-[(4-chloropyrimidin-2-yl)amino]benzonitrile). The starting materials 2-thioxo-2,3-dihydropyrimidin-4(1H)-one (**10**) and iodomethane were blended in the presence of NaOH base to attain compound **11**. Further, the nucleophilic substitution of **11** with *p*-aminobenzonitrile (**12**) in DME afforded compound **13**(iia). Finally, the target intermediate **3** was obtained by chlorinating the hydroxy group of **13** with POCl_3_ under reflux conditions. This route's advantage is that the reaction step is simple and short, and the total yield is up to 47%, and the disadvantage is that the reaction produces methanethiol, which is highly toxic and smelly. Complete elimination of this odour is highly challenging, which may increase the cost of large-scale industrial production. In addition, the boiling point of DME used in the reaction process is too high [15]. So it has been reported that the step (ii) using the melting method also has a higher yield and shorter reaction time [16, 17]. However, there is no report on the influence of temperature and reaction time on the yield, the optimal reaction temperature and reaction time cannot be determined.

**Scheme 4.**
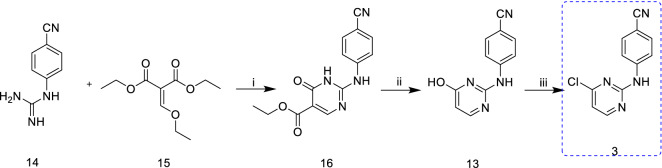
Synthesis of intermediate **3** from 1-(4-cyanophenyl)guanidine (**14**) and diethyl 2-(ethoxymethylene)malonate (**15**) as starting materials [18]. Reagents and conditions: (i) NMP, AcONa, 100 °C, 1 h; (ii) H_2_O, 155–160 °C; AcOH, 130–150 °C, 76%; (iii) POCl_3_, reflux, 20 min, 77%

Scheme 4 also illustrates the synthesis of intermediate **3**, and the cyclization of starting material 1-(4-cyanophenyl)guanidine (**14**) with substituted diethyl malonate (**15**) in the presence of NMP and sodium acetate afforded compound **16** with pyrimidine moiety. The subsequent reaction of **16** with water, acetic acid, leads to **13**, which further treated with POCl_3_ afforded the desired intermediate **3** by "one-pot method". Although it is a simple reaction with an overall yield of 58.5%, the starting material (**14**) is expensive, limiting the large-scale industrial application of this route [18].

**Scheme 5.**
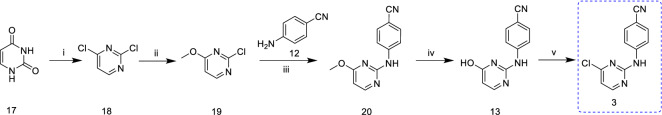
Synthesis of intermediate **3** from pyrimidine-2,4(1H,3H)-dione (**17**) as starting material [14]. Reagents and conditions: (i) C_6_H_5_N(CH_3_)_2_, POCl_3_, 0 °C; reflux, 4 h, 43%; (ii) CH_3_OH, NaOCH_3_, rt, 14 h, 50%; (iii) 2-Toluene sulfonic acid, dioxane, 100–110 °C, 14 h, 55%; (iv) Pyridine, 150–160 °C, 3 h, 87%; (v) POCl_3_, 0 °C, reflux, 1 h, 75%

In Scheme 5, uracil's reaction with POCl_3_ leads to the formation of 2,4-dichloropyrimidine (**18**), which was further treated with sodium methoxide in methanol afforded **19**. The acid-catalyzed nucleophilic addition between **19** and 4-aminobenzpnitrile in the presence of 2-toluenesulphonic acid in dioxane gave 4-[4-methoxypyrimidin-2-yl)amino]benzonitrile (**20**). The target intermediate **3** was achieved by reacting **20** with pyridine, followed by POCl_3_ under reflux condition. The reaction is relatively simple, but the process is too long and reducing the yields. Also, the highly toxic phosphorus oxychloride reagent is used repeatedly, which is harmful to the environment and public health. The entire route has a long reaction time and consumes a lot of energy, which is not suitable for industrial production [14].

### Synthesis of rilpivirine

**Scheme 6.**
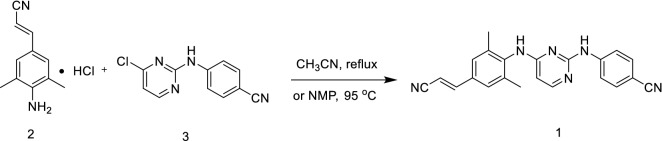
Synthesis of rilpivirine from intermediates **2** and **3** [19, 20]

Synthesis of the target compound rilpivirine (**1**) was achieved by allowing the reaction between (2*E*)-3-(4-amino-3,5-dimethylphenyl)prop-2-enenitrile hydrochloride (**2**) and 4-[(4-chloropyrimidin-2-yl)amino]benzonitrile (**3**) in acetonitrile under reflux condition for 69 h (Scheme 6, yield: 68.6%) [19]. This reaction's conditions are relatively simple, but the reaction time is longer, extending the industrial cycle production time and reducing the product's yield and quality. Also, it has been reported that NMP used as a solvent at 95 °C for 17 h to produce rilpivirine with 71.4% yield, wherein *cis-*isomer accounts for 0.7% [20]. However, it isn't easy to reclaim NMP in industrial production because of its higher boiling point.

There are many shortcomings in the synthetic strategy of rilpivirine. Notably, (a) intermediate **2** was mainly obtained by Heck reaction, and the catalyst palladium acetate and its ligands used in this reaction are expensive; (b) the starting material (**14**) used for the preparation of intermediate **3** is relatively expensive and the reaction temperature is too high; (c) when uracil is used as a starting material, the synthetic route in Scheme 5 is rather tedious, and the final reaction yield is too low; (d): the final step in the synthesis of rilpivirine, is too long (69 h) and causing energy consumption. Hence there is an urgent need to find more efficient and practical methods for synthesizing rilpivirine in the pharmaceutical industry. Herein, we represent our efforts to develop an efficient synthetic route with increased overall yield and reasonable reaction time.

## Results and discussion

**Scheme 7.**
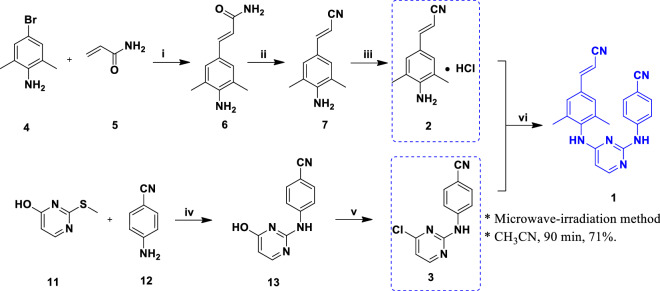
The improved synthetic procedure of rilpivirine. Reagents and conditions: (i) Pd(OAc)_2_, P(C_6_H_5_CH_3_)_3_, CH_3_COONa, DMAC, N_2_, 130 °C, 24 h, 51%; (ii) POCl_3_, 40 °C 8 h; (iii) EtOH, HCl, acetyl chloride, 0 °C, 30 min, total yield of steps (ii) and (iii) 95%; (iv) N_2_, 160 °C, 2 h, 180 °C, 4 h, 70%; (v) POCl_3_, reflux 8 h, 89%; (vi) CH_3_CN, 140 °C, 90 min, 71%

A novel synthetic procedure for the rilpivirine is successfully demonstrated in six steps with improved yield (21%). The method (Scheme 7) is developed to generate laboratory-scale rilpivirine without using toxic reagents (acrylonitrile), and high boiling point solvents (NMP). This method aims to develop an efficient synthetic route for intermediate **3** and improve the reaction conditions for the nucleophilic substitution reaction in the last step. To achieve the desired intermediate **3**, initially, a fusion reaction was carried out between 2-(methylthio)-4(3H)-pyrimidinone (**11**) and *p*-aminobenzonitrile (**12**) under an inert atmosphere, resulted in compound **13**. After that, compound **13** reacted with POCl_3_ under reflux conditions to yield the target intermediate **3**. As well as intermediate **2** was obtained as illustrated in Scheme 7. Finally, the nucleophilic substitution reaction between intermediates **2** and **3** through microwave-irradiation leads to the rilpivirine formation with improved yield (21%) and shorter reaction time (90 min).

### Optimization of synthesis steps and process parameters of intermediate **13**

The reaction for the synthesis of compound **13** was carried out through melting/fusion method with the temperature ranging from 160 °C to 200 °C. Herein, we investigated the effect of reaction temperature and reaction time on the yield of compound **13** (Table 1). During this process, the observations are: (1) when the reaction temperature was 160 °C, the reaction solution was completely solidified after 2 h, and the product yield was 64%; (2) when the temperature was 180 °C for 6 h, the product yield was 70%; (3) when the reaction temperature was 190 °C for 6 h, the product yield was 70.2%; (4) when the reaction temperature was 200 °C, no obvious increase in the yield was observed compared to the yield at 190 °C. To sum up, the reaction condition is selected to be 160 °C for 2 h, and then 180 °C for 4 h.
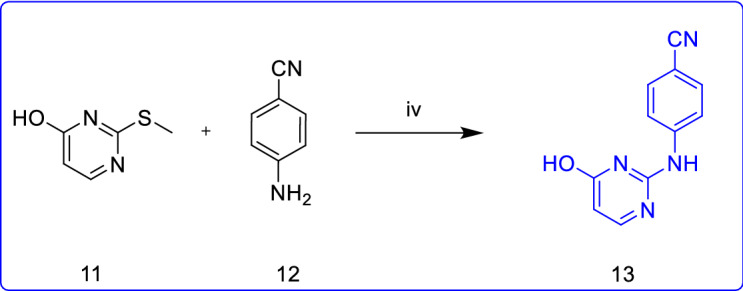

**Table 1.** Optimization of reaction temperature and time for compound **13** yield

Time (°C)	Time/h	Yield %
160	2	64
180	6	70
190	6	70.2
200	6	70.2

### Synthesis optimization of rilpivirine (**1**)

Considering the longer reaction time of the final step in the previous reports, we attempt to apply the microwave-assisted synthesis method in the amination step to shorten the reaction time and to improve the yield of rilpivirine. The orthogonal experiment investigated the effects of reaction solvent, temperature, pressure and reaction time on microwave-assisted amination yield. 
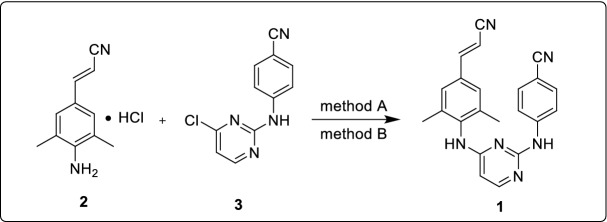


#### Optimization of reaction solvent

Among the methods reported in the literature, the most commonly used solvents are acetonitrile and NMP. In the preliminary study, we conducted the reaction under atmospheric pressure (method A). When we led our first attempt, dioxane, acetonitrile, and NMP solvents were used. It was observed that the reaction with acetonitrile solvent provided good yields, NMP with moderate to good and with dioxane the reaction is not formed. However, when we tried to use these three kinds of solvents for microwave reaction, the reaction results were not satisfactory. Table 2 shows that although the microwave reaction can proceed smoothly, the microwave reaction yield is still far from the traditional heating conditions.

**Table 2.** Optimization of solvent for the synthesis of rilpivirine with better yield

Solvents	Method	Temperature (°C)	Time	Yield%
Dioxane	A	90	12 h	0
CH_3_CN	A	reflux	69 h	69
NMP	A	90	16 h	75
Dioxane	B	100	30 min	0
CH_3_CN	B	100	60 min	40
NMP	B	100	60 min	43

*Method* *A* Under atmospheric pressure, *Method B* Microwave-assisted synthesis

Considering that different solvents are used as the reaction, none of the reactions was ideal. We speculated that the reaction temperature and time have a significant influence on the reaction yield. Since the acetonitrile yield as solvent was similar to NMP and considering industrial production cost, we choose low-boiling acetonitrile as the reaction solvent for further exploration. Therefore, a single variable method was applied to investigate the effect of reaction temperature and time on yield. The results are shown in Table 3.

**Table 3.** Optimization of reaction temperature and time

Temperature (°C)	Time (min)	Yield (%)
120	60	48
120	90	51
120	120	56
130	60	52
130	90	60
130	120	65
140	60	60
140	90	71
140	120	71.4
150	60	65
150	90	71.2
150	120	70

We can conclude that the yield was improved with the increased reaction time and temperature. But there is a decreasing tendency of the yield when the temperature is 150 °C and reaction time is more than 120 min. Accordingly, the amination reaction's optimized conditions were determined as follows: microwave-irradiation reaction with acetonitrile solvent at 140 °C for 90 min produced the amination product with 71% yield, which was higher than that of the traditional method (69%).

## Conclusion

In conclusion, we provide a synthetic method for rilpivirine, an essential drug in the treatment of acquired immunodeficiency syndrome (AIDS). The experiment mainly optimized the route of rilpivirine and the key process parameters of each step by controlling the single variable method to determine the optimal experimental conditions. The total yield was improved from 18.5 to 21%, and the reaction time of the last step decreased from 69 h to 90 min. The details are as follows: (1) synthesis of 4-((4-hydroxypyrimidin-2-yl)amino)benzonitrile (**13**) the reaction was carried out at 160 °C for 2 h, and then the temperature was raised to 180 °C for 4 h. After the treatment, a small amount of *N,N*-dimethylformamide was added to dissolve the residues, subsequently, the mixture was filtered and washed with acetonitrile to give the product with 70% yield; (2) the total yield of the intermediate **3** increased from 52 to 62% compared to the original patent. The total synthesis yield of the intermediate **2** and rilpivirine is equivalent to original patents. (3) For the rilpivirine synthetic step, a microwave-irradiation method is selected instead of the traditional method in the original patent, and the yield of amination reaction is increased to 71%. Therefore, the total synthetic yield of rilpivirine risen from 18.5 to 21%. The method proceeded in six linear steps with a gram scale and did not use high toxicity starting materials and high boiling-point solvent. This efficient and environmental-friendly process and the optimum conditions for rilpivirine preparation may form the basis for future manufacturing route.

## Experimental section

Thin-layer chromatography (TLC) was performed on Silica Gel GF254 for TLC (Merck), and spots were visualized by irradiation with UV light (λ = 254 nm). ^1^H NMR and ^13^C NMR spectra were recorded on a Bruker AV-400 NMR spectrometer or a Bruker Avance-600 NMR spectrometer using solvents as indicated. Chemical shifts were reported in *δ* values (ppm) with tetramethylsilane as the internal reference, and *J* values were reported in hertz (Hz). Melting points (m.p.) were determined on a micro melting point apparatus (TianJin Analytical Instrument Factory, Nankai, Tianjin, China). Mass spectra were recorded on an LC Autosampler device: Standard G1313A instrument. The microwave reaction was conducted on a CEM Discover (0–600 W, 2450 MHz) instrument and the conventional high-pressure reaction was performed on Parr 4590 device. Rotary evaporators were served in the concentration of the reaction solutions under reduced pressure.

### (*E*)-3-(4-amino-3,5-dimethylphenyl)acrylamide (**6**)

4-Bromo-2,6-dimethylaniline **4** (50 mmol, 10 g), acrylamide **5** (50 mmol, 3.7 g), sodium acetate (75 mmol, 6.15 g), palladium acetate (15 mmol, 3.4 g) and tris(2-methylphenyl)phosphine (15 mmol, 4.6 g) was placed in a round bottom flask, and *N*,*N*-dimethylacetamide was slowly added to the round bottom flask under nitrogen, then the mixture was heated to 130 °C for 24 h. After the completion of the reaction, the mixture was cooled to room temperature, filtered over celite, evaporated. The residue was recrystallized in ethyl acetate and petroleum ether to give the product (*E*)-3-(4-amino-3,5-dimethylphenyl)acrylamide (**6**), 4.8 g, yield: 51%.

### (*E*)-3-(4-amino-3,5-dimethylphenyl)acrylonitrile hydrochloride (**2**)

To a round-bottomed flask consisting of 10 mL of phosphorus oxychloride was added compound **6** slowly at 0 °C and then refluxed for 8 h at 40 °C. After completion of the reaction was monitored by TLC, and the reaction mixture was slowly poured into the ice-water mixture and stirred for 1 h and then filtered to obtain the crude (*E*)-3-(4-amino-3,5-dimethylphenyl)acrylonitrile (**7**) as white crystal solid.

The obtained crude product **7** was dissolved in anhydrous ethanol, and then added ethanolic hydrochloric acid solution, and stirred for 30 min, then filtered to obtain (*E*)-3-(4-amino-3,5-dimethylphenyl)acrylonitrile hydrochloride (**2**), 5.0 g. Yield: 95%. mp: 78–80 °C. ^1^H NMR (400 MHz, CDCl_3_): *δ* 7.22 (d, *J* = 16.5 Hz, 1H, CH=), 7.05 (s, 2H, Ph-H), 5.59 (d, *J* = 16.5 Hz, 1H, =CH), 3.95 (s, 2H, NH), 2.18 (s, 6H, CH_3_). ^13^C NMR (100 MHz, CDCl_3_): *δ* 150.94, 146.21, 129.86, 128.02, 123.28, 121.50, 119.60, 90.28, 77.38, 77.06, 76.74, 17.50. ESI-MS: *m/z* 173.54 [M+H]^+^, C_11_H_12_N_2_ (172.10).

### Preparation of ethanolic hydrochloric acid solution

The solvent of anhydrous ethanol was stirred under ice bath, and then 41 mL of acetyl chloride reagent added slowly dropwise and stirred for 30 min to obtain an ethanolic hydrochloric acid solution.

### 4-((4-hydroxypyrimidin-2-yl)amino)benzonitrile (**13**)

Starting materials 2-methylthio-4-pyrimidinone **11** (70 mmol, 10 g) and *p*-aminobenzonitrile **12** (77 mmol, 9.14 g) were taken together, stirred under the inert condition at 160 °C for 2 h and then increased the temperature to 180 °C for 4 h. Then the reaction was allowed to room temperature and added an appropriate amount of *N, N*-dimethylformamide. The resulted mixture was kept in the ultrasonic vibrator for 30 min. Then the mixture was filtered and washed with acetonitrile and dichloromethane, dried in vacuo to give 4-((4-hydroxypyrimidin-2-yl)amino)benzonitrile (**13**) as a white solid (10.3 g, yield 70%).

### 4-((4-chloropyrimidin-2-yl)amino)benzonitrile (**3**)

Compound **13** was dissolved in 20 mL of phosphorus oxychloride at 0 °C and then refluxed for 1h. After completion, the mixture was cooled to room temperature, slowly poured into 100 g of crushed ice and stirred for half an hour. After filtration, the obtained filter cake was dissolved in 50 mL of water, and the pH was adjusted to 7–8 with potassium carbonate and filtered. The filtrate was washed with acetonitrile and dried to obtain 4-((4-chloropyrimidin-2-yl)amino)benzonitrile (**3**) as a white solid (9.9 g). Yield: 89%. mp: 209-210 °C. ^1^H NMR (400 MHz, DMSO-*d*_*6*_): *δ* 10.58 (s, 1H), 8.55 (d, *J* = 5.2 Hz, 1H, C6-pyrimidine-H), 7.87 (dd, 4H, Ph-H), 7.13 (d, *J* = 5.2 Hz, 1H, C6-pyrimidine-H); ESI-MS: *m/z* 231. 2 [M+H]^+^, C_11_H_7_ClN_4_ (230.04).

### Rilpivirine (**1**)

A mixture of compound **2** (1 g, 4.8 mmol) and **3** (1.2 g, 5.2 mmol) along with 10 mL acetonitrile was transferred into 20 mL microwave vial and set the reaction temperature at 140 °C, 2 h. After completion, the reaction mixture was cooled to room temperature and then 10% potassium carbonate solution was added dropwise to the vial to adjust the pH to 8–9. The obtained filtrate was washed with acetonitrile to obtain the target molecule rilpivirine as a white solid (1.28 g). Yield 71%. mp: 138–140 °C. ^1^H NMR (400 MHz, DMSO-*d*_*6*_): *δ* 9.62 (s, 1H, NH), 8.95 (s, 1H, NH), 7.98 (t, *J* = 27.2 Hz, 2H, Ph-H), 7.69 (m, 3H, =CH, Ph-H), 7.49 (s, 2H, Ph-H), 6.46 (d, *J* = 16.7 Hz, 1H, =CH), 2.18 (s, 6H, CH_3_). ESI-MS: *m/z* 367.24 [M+H]^+^, C_22_H_18_N_6_ (366.16).

## Data Availability

All data are fully available without restriction.

## Abbreviations

RPV: Rilpivirine
HIV-1: Human immunodeficiency virus 1
RT: Reverse transcriptase
NNRTIs: Non-nucleoside reverse transcriptase inhibitors
HAART: Highly active antiretroviral therapy
DME: Dimethoxyethane
NMP: *N*-methylpyrrolidin-2-one
AIDS: Acquired immunodeficiency syndrome
mp: Melting-point
TLC: Thin layer chromatography
MS: Mass spectra
^1^H NMR: ^1^H-Nuclear magnetic resonance
^13^C NMR: ^13^C-Nuclear magnetic resonance
